# Genetic ablation of metabotropic glutamate receptor 5 in rats results in an autism-like behavioral phenotype

**DOI:** 10.1371/journal.pone.0275937

**Published:** 2022-11-16

**Authors:** Adrien A. Eshraghi, Idil Memis, Florence Wang, Isaiah White, Emily Furar, Jeenu Mittal, Moeed Moosa, Coleen M. Atkins, Rahul Mittal

**Affiliations:** 1 Hearing Research and Communication Disorders Laboratory, Department of Otolaryngology, Neurotology Division, University of Miami Miller School of Medicine, Miami, Florida, United States of America; 2 Department of Pediatrics, University of Miami Miller School of Medicine, Miami, Florida, United States of America; 3 Department of Neurological Surgery, University of Miami Miller School of Medicine, Miami, Florida, United States of America; University of Minnesota Twin Cities, UNITED STATES

## Abstract

Autism spectrum disorder (ASD) is a neurodevelopmental disorder characterized by deficits in communication, and social skills, as well as repetitive and/or restrictive interests and behaviors. The severity of ASD varies from mild to severe, drastically interfering with the quality of life of affected individuals. The current occurrence of ASD in the United States is about 1 in 44 children. The precise pathophysiology of ASD is still unknown, but it is believed that ASD is heterogeneous and can arise due to genetic etiology. Although various genes have been implicated in predisposition to ASD, metabotropic glutamate receptor 5 (mGluR5) is one of the most common downstream targets, which may be involved in autism. mGluR5 signaling has been shown to play a crucial role in neurodevelopment and neural transmission making it a very attractive target for understanding the pathogenesis of ASD. In the present study, we determined the effect of genetic ablation of mGluR5 (*Grm5*) on an ASD-like phenotype using a rat model to better understand the role of mGluR5 signaling in behavior patterns and clinical manifestations of ASD. We observed that mGluR5 *Ko* rats exhibited exaggerated self-grooming and increased marble burying, as well as deficits in social novelty. Our results suggest that mGluR5 *Ko* rats demonstrate an ASD-like phenotype, specifically impaired social interaction as well as repetitive and anxiety-like behavior, which are correlates of behavior symptoms observed in individuals with ASD. The mGluR5 *Ko* rat model characterized in this study may be explored to understand the molecular mechanisms underlying ASD and for developing effective therapeutic modalities.

## Introduction

Autism spectrum disorder (ASD) is a neurodevelopmental disorder characterized by deficits in behavior and communication [1–4]. The current occurrence of ASD in the United States is about 1 in 44 children [5]. There are three core symptoms of ASD: impaired social behavior, stereotypic/repetitive behaviors, and sensory/communication deficits [1, 6–8]. The commonly observed behavioral disturbances also include aberrant sensitivity to sensory stimulations, hyperactivity, and possible self-injury [1].

ASD is a complex disorder, and a wide arsenal of factors have been involved in the pathophysiology of this neurodevelopmental disorder. Therefore, a multidisciplinary approach is the key to understand its etiology and for the design of rational interventions. Studies in animal models are aimed at simulating the core phenotypes associated with ASD to identify the mechanisms that underscore the entire spectrum of the disorder [9–15]. Due to the lack of suitable ASD models, the exact mechanisms by which ASD develops are still unknown. Rat models may be more appropriate than mice for understanding ASD pathogenesis, as rats exhibit complex social behavior especially during development [16–21]. Mouse play behavior during development is less conspicuous and comprises few interaction elements. In contrast, normal young rats are playfully aggressive creatures, wrestling, boxing, and pinning their siblings down by the neck unlike mice. This is important since ASD is a developmental disorder and modeling the impaired social behavior during development is critical in clinically relevant animal models of ASD. In addition, as compared to mice, rats use a rich acoustic communication system [22–27]. All these findings suggest that the rat may be a more suitable animal model as compared to mice for understanding the molecular mechanisms underlying ASD etiology.

Although the exact etiology of ASD remains an enigma, at least 30% of cases have an underlying genetic etiology [28–35]. The most common gene variants involved in ASD include *SHANK3*, *MECP2*, *NLGN3*, *NRXN1* and *FMR1* [36–50], some of which are regulated by the metabotropic glutamate receptor (mGluR) pathway, especially mGluR5, thus making it a very attractive target for understanding the pathogenesis of ASD [51]. mGluR5 is a seven-transmembrane spanning G-protein coupled receptor (GPCR) located in the postsynaptic membrane of excitatory synapses, neuronal nuclear membranes, glia, and oligodendrocytes [52–56]. mGluR5 is important for neuronal-glial communication and in neuronal homeostasis including the control of glutamate release and uptake by astrocytes [57]. mGluR5 signal transcription events occur either via phospholipase-C (PLC) to act ultimately on the mitogen activated protein kinase (MAPK) and extracellular signal-regulated kinase (ERK), or via phosphoinositide-3-kinase (PI3K) and the mammalian target of rapamycin (mTOR) [58].

mGluR5 has been shown to play an important role in the pathophysiology of ASD by regulating the function of a number of proteins involved in synaptic transmission, including Shank3 [51]. As a proof of this concept, pharmacological enhancement of mGluR5 has been shown to rescue behavioral deficits in *SHANK3* knockout (*Ko*) mice [59]. mGluR5 and Shank3 interact with each other primarily through homer proteins [51]. In addition, the gene expression of *mGluR5* was significantly decreased in ASD patients versus control human subjects in a post-mortem brain stereological investigation [60]. The intensity of the staining of mGluR5-positive neurons was also significantly decreased in ASD versus control subjects [60]. The single nucleotide polymorphisms in *Grm5*, the gene that encodes mGluR5, have been found to be a predictive genetic classifier for ASD [61]. The mGluR5 antagonists administered to wild-type (WT) rats have been shown to impair social interaction, which is a core clinical deficit in ASD [62]. Further, reduced mGluR5 expression has been observed in *Mecp2 Ko* mice as well as in the motor cortex of autopsy samples from Rett syndrome (RS) patients [63]. RS is a neurodevelopmental disorder that results from *de novo* mutations in the *MECP2* gene and shares many symptomatic, as well as pathological commonalities with ASD [64, 65]. Additionally, treatment of *Mecp2*-deficient mice with mGluR5 positive allosteric modulator (PAM), VU0462807, improves behavior defects [63]. These basic science and clinical studies employing ASD individuals strongly lay the foundation for the usefulness and relevance of mGluR5 *Ko* rats as suitable models of ASD. The availability of a preclinical mGluR5 rat model that is a downstream target molecule for some of the genes implicated in ASD will provide a unique tool to understand the underlying molecular mechanisms behind the etiology of ASD. This model will help us to comprehend the neurodevelopmental changes that underlie the behavioral deficits observed in ASD and will open the doors for evaluating the efficacy of future therapeutic interventions.

## Methods

### Animals

Heterozygous breeders of mGluR5 *Ko* rats on Sprague Dawley background were obtained from the Envigo company (Indianapolis, IN, USA). The model was generated by a biallelic deletion of the metabotropic glutamate receptor 5 (mGluR5 or *Grm5*). All experimental animals were obtained from heterozygous crossings. Male and female rats were used in these experiments. In total, 36 rats [19 WT (9 males, 10 females) and 16 *Ko* (8 males and 8 females) animals] were subjected to a comprehensive battery of ASD-associated behavior tests at 12 weeks of age. Animals were group housed in a room with 12-hr light /12-hr dark Light/Dark cycle. Food and water were provided *ad libitum*. To control potential litter effect, one animal each of WT and *Ko* per litter was randomly selected for behavior phenotyping. This experimental design allows the use of standard statistical methods, such as *t* tests for analysis [66, 67]. The rest of the animals were used in additional different experiments. The animals for this study were derived from 19 pregnant dams as heterozygous breeding scheme was used providing both WT and *Ko* rats. Genotyping was performed by Transnetyx company (Cordova, TN, USA) using custom designed probes having primer sequence, mGluR5 F CTTCATGAGGGTTGTACCTTCC; mGluR5 R GTGTGCACAGCTGAGACATAAG. Behavioral analyses were conducted by the trained observers that were blinded to the rat genotype. The study protocol was approved by the Animal Care and Use Committee of the University of Miami and was in full compliance with the NIH guidelines for the care and use of laboratory animals.

### Open field (OF)

Rats were brought to the testing room at least 30 minutes before the test. Each animal was videotaped for 10 minutes undisturbed after a 20-minute habituation time in their own cage (46 cm length × 23.5 cm wide × 20 cm high). The dimension of the open field (OF) chamber was 85 x 50 x 50 cm. Animals were placed in the middle of the arena at the start of the test. They remained in the arena for 10 minutes and their positions were tracked using Ethovision Version 11.5 (Noldus Information Technology, Netherlands). The center region was defined as an area that covered 42.35% of the total arena (artificial dimension: 60 x 30 cm inside vs. 85 x 50 cm total arena). Time spent in the center (center duration, in sec) and in the periphery of the arena (periphery duration, in sec) as well as the proportion of time spent in the perimeter of the box was used as an index of anxiety [68–70]. We also determined the total distance traveled (in cm) as an index of locomotor activity. In addition, the rearing frequency was used as a measure of exploratory behavior and anxiety [71, 72].

### Self-grooming

After a 20-minute habituation period, animals were videotaped undisturbed for 10 minutes. The number of bouts of grooming sessions and the time spent in grooming was determined by two trained observers who were blinded to the experimental conditions. Self-grooming behaviors included wiping the nose, face, head, and ears with forepaws, as well as licking the body, anogenital area and tail [68, 73, 74]. The influence of rat odors was prevented by thoroughly cleaning the cage at the beginning of each trial.

### Social interaction test

The social interaction test was performed using a three-chamber compartment comprising of three phases. In the first phase, the test animal was introduced to the middle compartment of the three- chambered apparatus and was allowed 5 minutes to freely explore left and right chambers each containing an empty Plexiglas cage. In the second phase, a non-familiar wild-type rat matched for sex and age (“stranger rat”) was placed in the cage in the right compartment. The test rat was placed in the middle chamber with closed connecting doors. The experiment started when the operator opened the doors, and the rat behavior was recorded over 10 minutes. Sociability was assessed by recording the time spent in the “stranger rat” chamber (in seconds), and in the empty cage chamber (in seconds) [70, 73, 75, 76].

Immediately following the second phase of the test, a novel wild-type rat matched for sex and age (“novel rat”) was placed in the cage in the left chamber while the “stranger rat” (from the second phase of the test) became familiar (“familiar rat”) to the test animal in this third phase. The search for social novelty was assessed by recording the time spent in the “familiar rat” chamber (in seconds) and in the “novel rat” chamber (in seconds) [70, 73, 75, 76].

### Marble-burying test (MBT)

MBT was used to measure repetitive and anxiety-related behaviors [77, 78]. The test rat was left undisturbed for one hour in the testing room in its home-cage for acclimation. In five cm high fresh bedding, 20 marbles (previously washed with 90% alcohol) were placed equally distant in the testing area. After the acclimation period, the test animal was allowed to bury marbles freely for 30 minutes. At the end of the test, the number of marbles buried was counted. Marbles were considered buried if more than two-thirds of their height was covered with the bedding. The marbles were thoroughly cleaned after each experiment followed by replacement of new bedding in the testing chamber. Experiments were also video-recorded, and animal’s cumulative time spent digging was manually scored with a stopwatch by two trained observers who were blinded to the experimental conditions.

### Statistical analysis

Statistical analyses were conducted with XLSTAT and GraphPad Prism version 28. Quantitative variables were compared using a Student’s *t*-test. A Mann-Whitney’s test was performed for nonparametric samples. The threshold of statistical significance was set with a *p* < 0.05.

## Results

### Exaggerated self-grooming behavior in mGluR5 Ko rats

**Fig 1:**
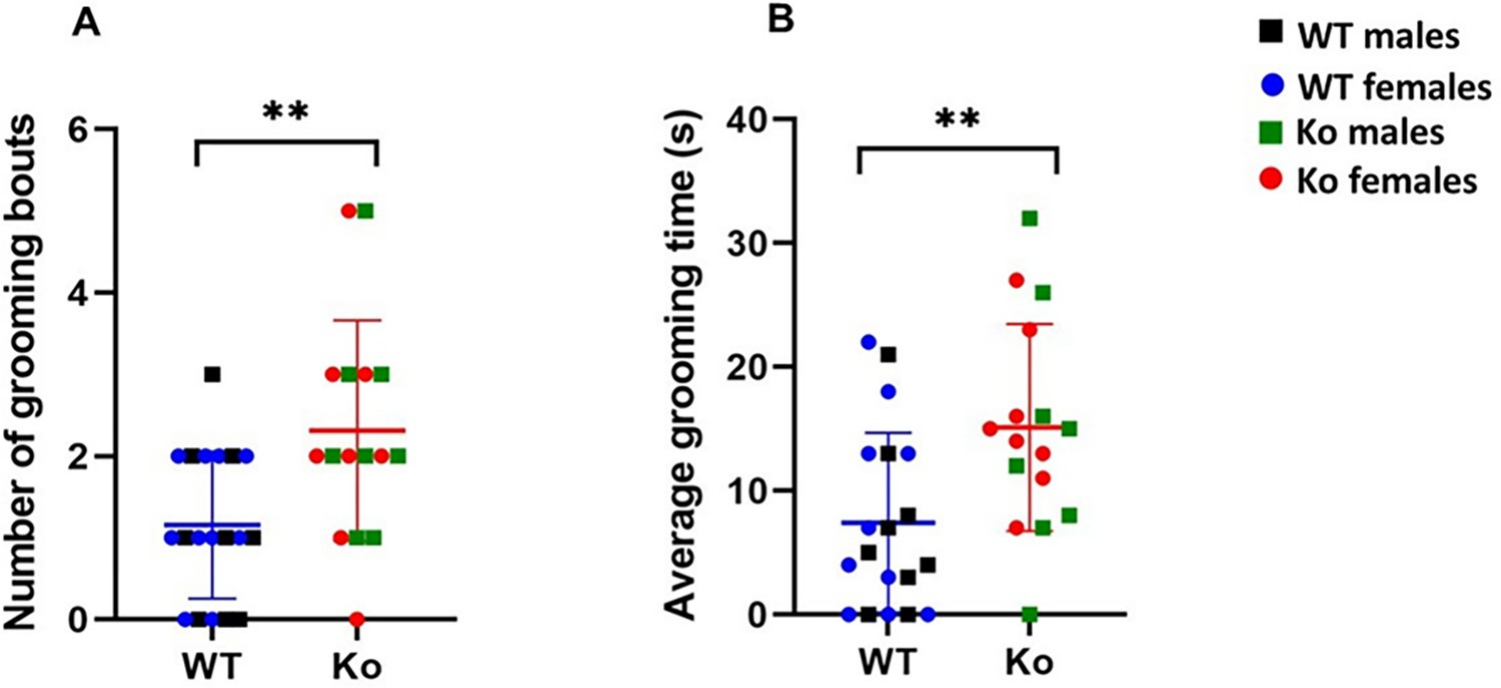
mGluR5 *Ko* rats show exaggerated self-grooming. The *Ko* rats groomed significantly more frequently (**A**) and for longer durations (**B**) compared to the WT animals. **p < 0.01 *Ko* versus WT animals.

Excessive self-grooming has been observed in preclinical animal models of ASD representing repetitive behaviors and as expression of anxiety [79, 80]. We observed that the *Ko* rats self-groomed significantly more frequently and for longer durations compared to the WT animals (*p* < 0.01) (**Fig 1A and 1B**). The mean grooming frequency was 3.31 ± 1.35 for the *Ko* rats compared to 1.16 ± 0.89 for the WT rats. Average grooming time was 15.25 ± 8.37 seconds and 7.42 ± 2.25 seconds in the *Ko* and WT rats, respectively. However, there was no statistically significant difference in grooming frequency and grooming time between *Ko* males and *Ko* females (*p* > 0.05).

### Increased rearing frequency in mGluR5 Ko rats

**Fig 2:**
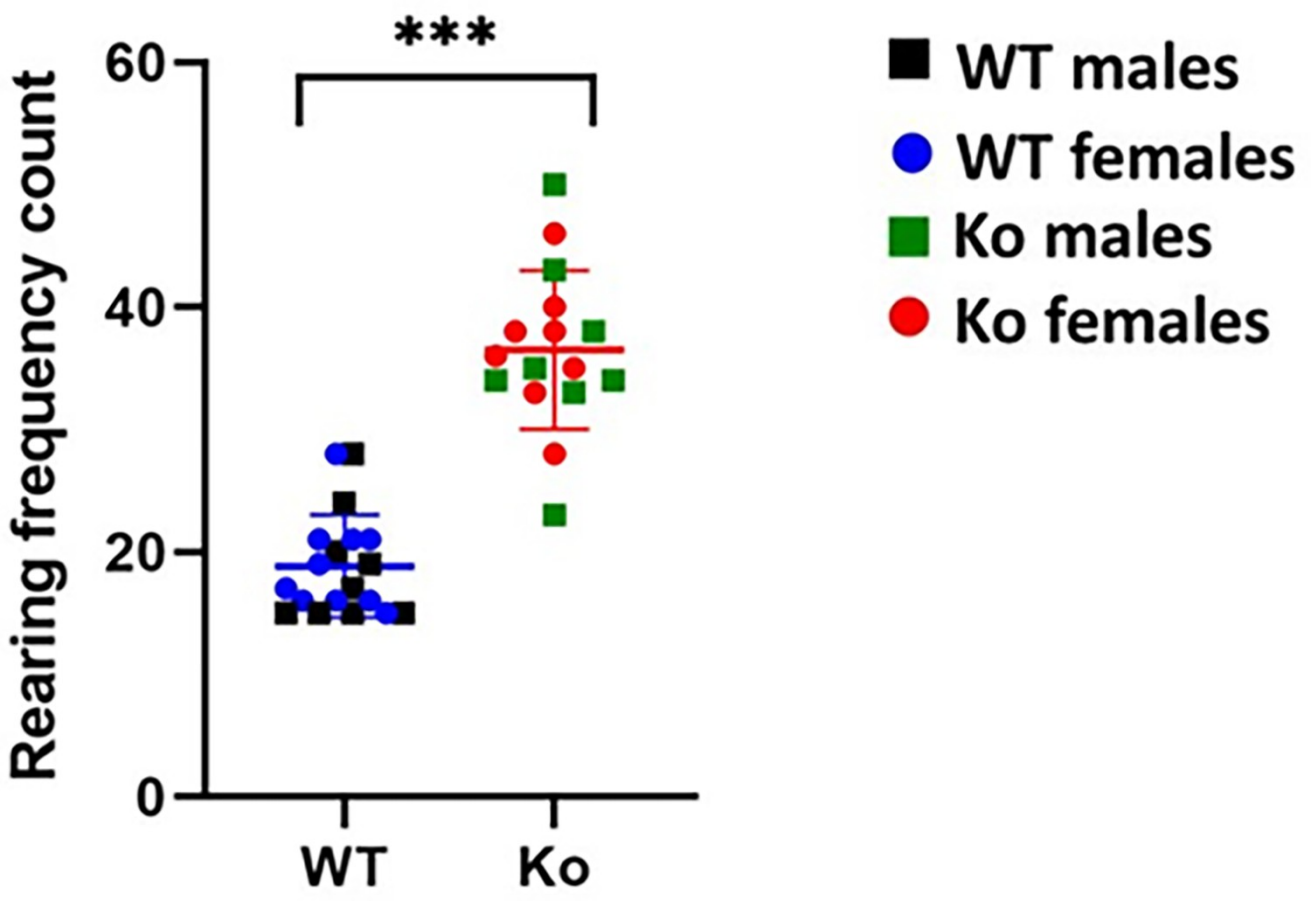
Rearing frequency is increased in mGluR5 *Ko* rats. The frequency of rearing was significantly higher in the *Ko* group than in the *WT* rats suggesting anxiety-like phenotype. ***p < 0.001 *Ko* versus WT animals.

Rearing frequency is the frequency with which the rodent stands on its hind legs in the open field, which might be a direct measure of anxiety [71, 72, 81, 82]. We observed that the frequency of rearing was significantly higher in the *Ko* group than in the *WT* rats (*Ko*: *Mean* = 36.50, *SD* = 6.48; *WT*: *Mean* = 18.84, *SD* = 4.18; *p* < 0.001) (**Fig 2**). We did not find any statistically significant difference in rearing frequency between *Ko* males and *Ko* females (*p* > 0.05).

### Increased marble-burying behavior and digging time in mGluR5 Ko rats

**Fig 3:**
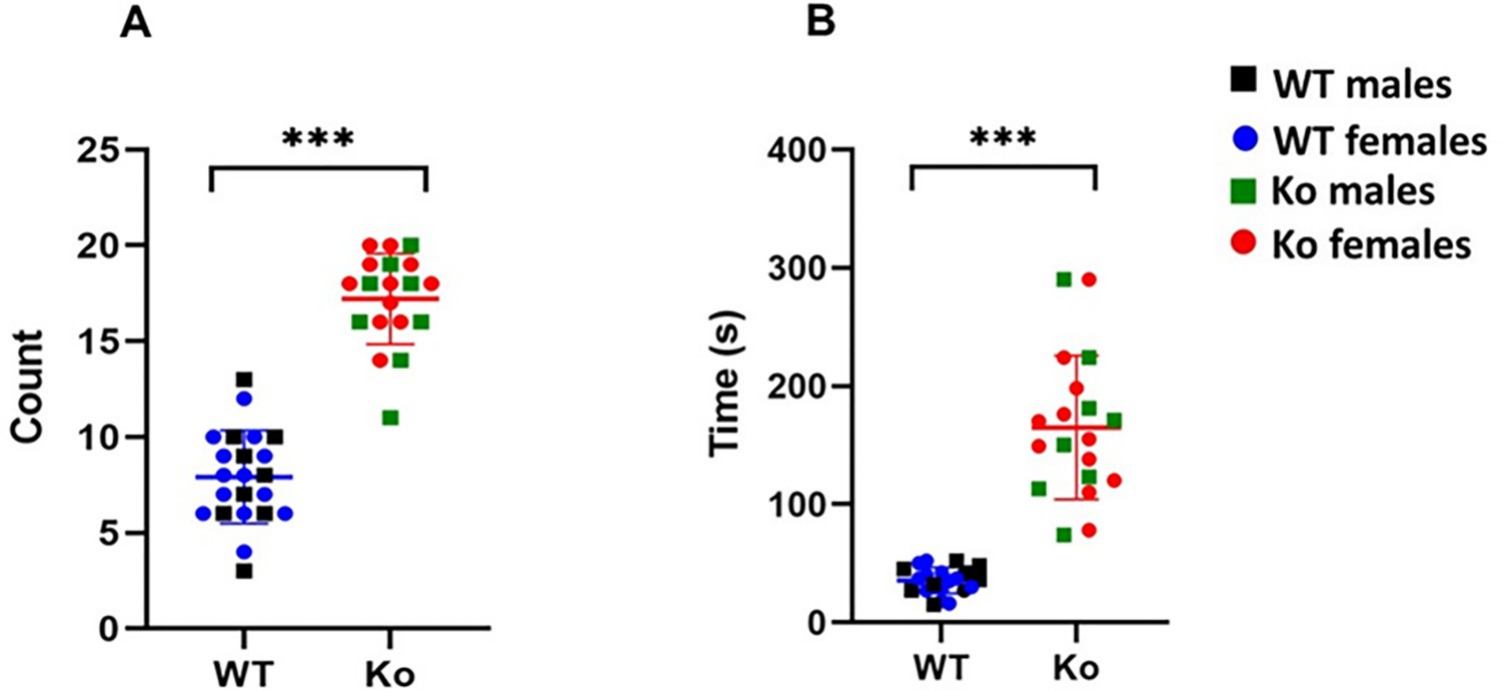
mGluR5 *Ko* rats show marble burying phenotype related with ASD-like phenotype. A. The *Ko* rats significantly buried more marbles compared to the WT animals. B. The total digging time was significantly higher in the *Ko* rats compared to the WT animals. ***p < 0.001 *Ko* versus WT animals.

The MBT allows in assessing anxiety-like, and repetitive behaviors. The effect of genetic ablation of mGluR5 (or *Grm5*) on MBT in a rat model is still not known. Therefore, we subjected WT and *Ko* rats to MBT. We determined the number of marbles buried as well as the total digging time in *Ko* and WT rats. We observed that the *Ko* rats significantly buried more marbles compared to the WT animals *(Ko*: *Mean =* 17.21, *SD* = 0.52; *WT*: *Mean* = 7.91, *SD* = 0.48; *p* < 0.001) (**Fig 3A)**. In addition, the total digging time was significantly higher in *Ko* rats compared to WT animals *(Ko*: *Mean =* 169.95, *SD* = 9.48; *WT*: *Mean* = 35.41, *SD* = 8.33; *p* < 0.001) (**Fig 3B).** These results suggest that the *Ko* rats exhibit repetitive behavior and are more anxious than the WT animals, which are the hallmarks of ASD-like phenotype. However, there was no statistically significant difference between *Ko* males and *Ko* females (*p* > 0.05).

### Open field test

**Fig 4:**
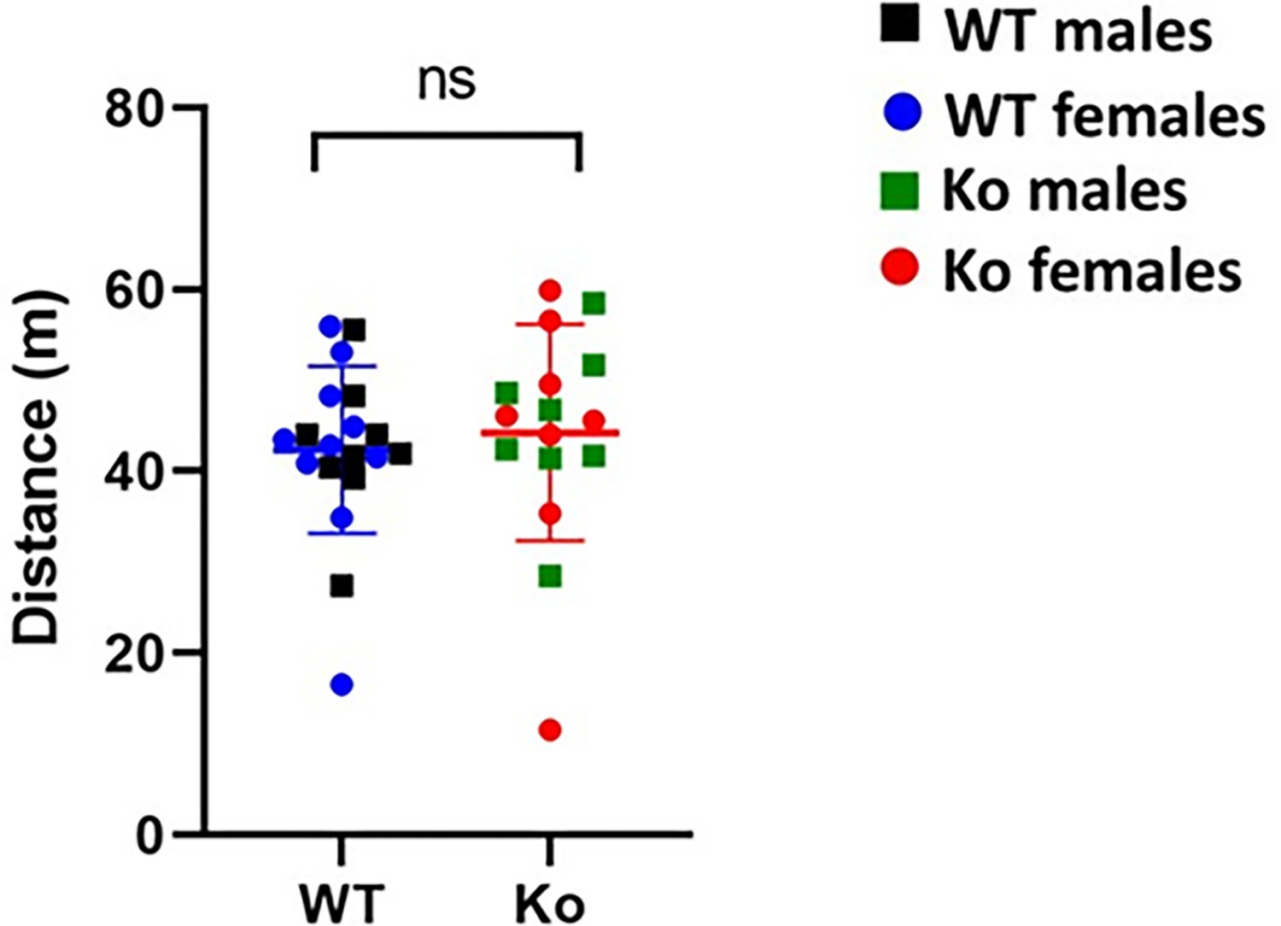
mGluR5 *Ko* rats do not show impaired locomotion. There was no significant difference in locomotor activity of the *Ko* rats compared to the WT animals. *p* > 0.05 *Ko versus* WT animals. ns: non-significant.

**Fig 5:**
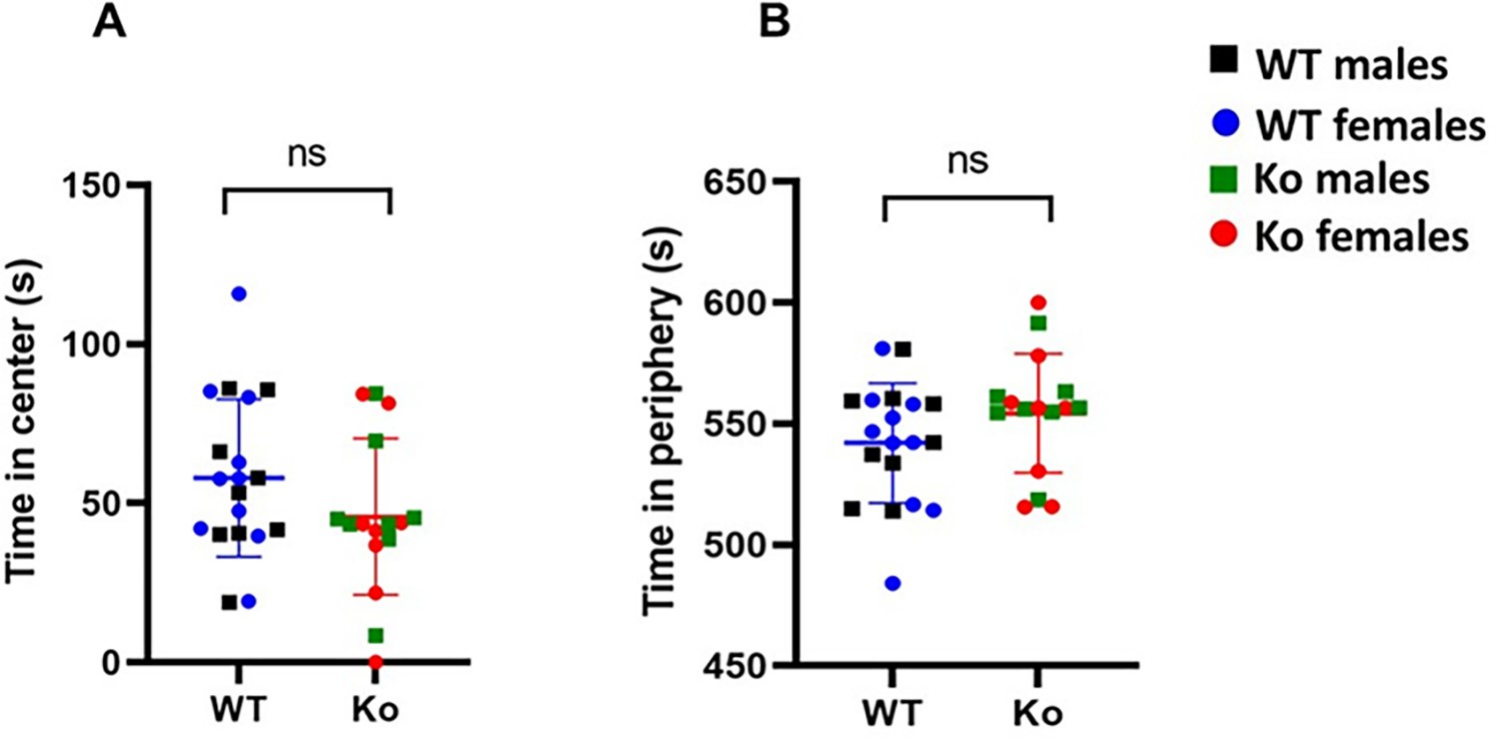
Perimeter preference in open-field test. The mGluR5 *Ko* rats do not show differences in time spent in center (**A**), and in perimeter (**B**) compared to WT animals. *p* > 0.05 *Ko* versus WT animals. ns: non-significant.

The open-field test is used to measure locomotor activity and anxious behavior of animal models. We observed that there was no significant difference in the total distance travelled between the *Ko* and WT rats (*Ko*: *Mean* = 44.26, *SD* = 11.91; *WT*: *Mean* = 42.38, *SD* = 9.19; *p* = 0.60) (**Fig 4**). These results suggest that were no significant differences in the locomotor activity of *Ko* and WT rats. We then determined time spent in the center and periphery by the *Ko* and WT rats (**Fig 5A and 5B**). There was no statistical difference between the *Ko* and WT rats in time spent in periphery and center (*p* > 0.05).

### mGluR5 Ko rats exhibit deficits in sociability and social novelty

**Fig 6:**
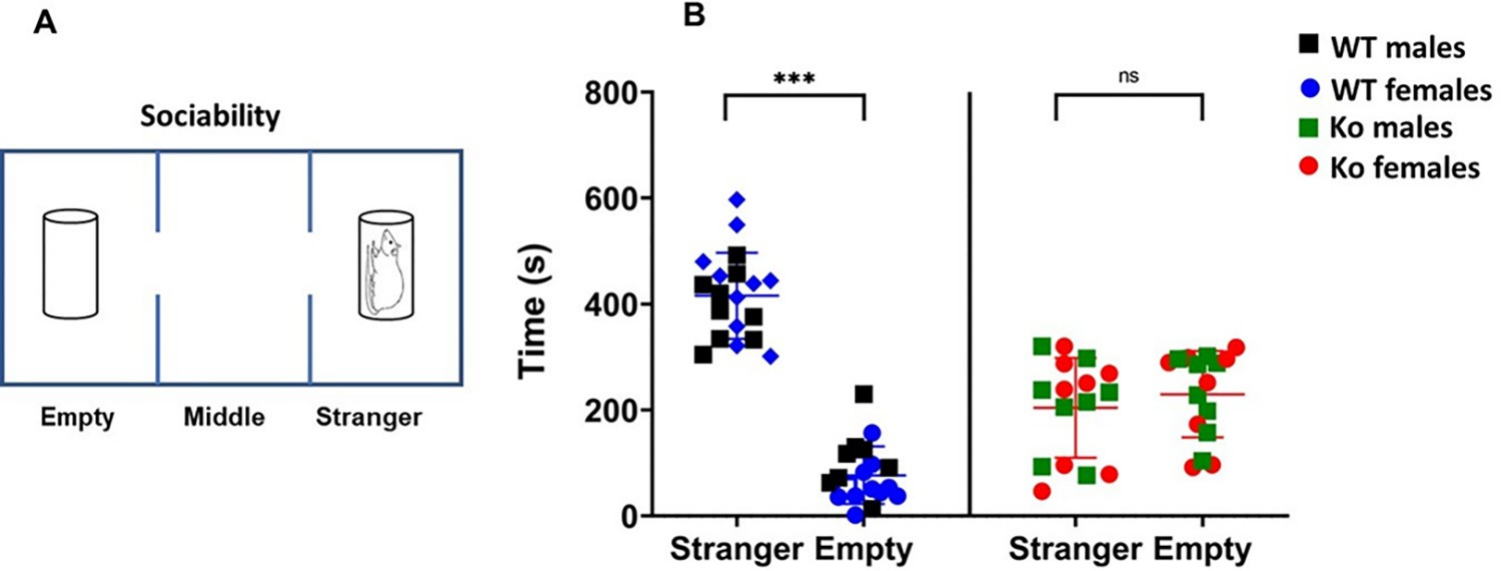
Three chamber sociability test. **A)** Schematic representation of three-chamber sociability test. **B)** The mGluR5 *Ko* rats do not exhibit sociability as there was no statistically significant difference between time spent with the stranger rat over the empty cage (*p* > 0.05). On the other hand, WT rats preferred spending time with the stranger rat compared to the empty cage (***p < 0.001). ns: non-significant.

**Fig 7:**
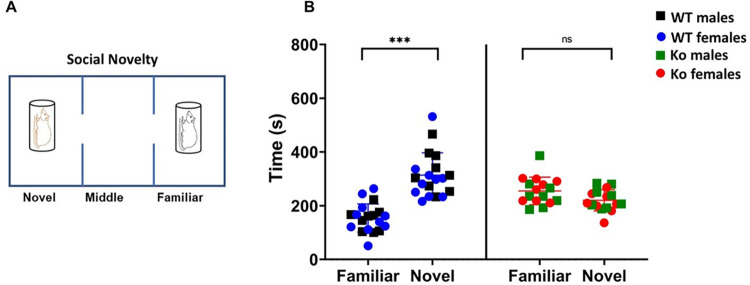
mGluR5 *Ko* rats exhibit no preference for social novelty. **A)** Schematic representation of social-novelty test. **B)** WT rats showed the preference for social novelty as they spent significantly more time with novel rat compared to the familiar rat (***p < 0.001). On the other hand, the mGluR5 *Ko* rats showed no preference and there was no statistically significant difference in total time spent with the novel rat over the familiar rat suggesting reluctance to social novelty (*p* > 0.05). ns: non-significant.

Using the three-chamber test, we assessed the sociability and social novelty behaviors (**Figs 6A** and **7A**). We observed that there was a statistically significant difference in sociability between *Ko* and WT rats. The WT rats spent significantly more time in the stranger rat chamber compared to the empty chamber (**Fig 6B**) (*p* < 0.001). On the contrary, the *Ko* rats showed no preference for stranger rat and empty (*p* > 0.05). These findings indicate that *Ko* rats show deficits in sociability. However, there was no statistically significant difference in sociability between *Ko* males and *Ko* females (*p* > 0.05).

During the third phase of the three-chambers test assessing the search for social novelty, a novel rat age and sex-matched (“novel rat”) was inserted in the cage of the left chamber while the “familiar rat” remained in the right chamber (**Fig 7A**). The WT rats spent more time in the novel rat chamber compared to time spent in the familiar rat chamber (*p* < 0.001). On the other hand, *Ko* rats showed no preference and there was no statistically significant difference in time spent in familiar and novel rat chambers (*p >* 0.05) (**Fig 7B**). These results suggest the reluctance to social novelty of *Ko* rats as compared to WT animals. However, there was no statistically significant difference in social reluctance between *Ko* males and *Ko* females (*p* > 0.05).

## Discussion

Metabotropic glutamate receptor mediated signaling, especially through mGluR5, has been hypothesized to play a crucial role in the pathophysiology of ASD. In support of this concept, the pharmacological enhancement of mGluR5 ameliorates behavioral deficits in preclinical animal models of ASD [59]. As mGluR5 can play an important role in predisposition to ASD, it is worthwhile to examine the effects of genetic deletion of mGluR5 (or *Grm5*) on ASD-associated behavioral manifestations. In the present study, we subjected mGluR5 *Ko* rats to a battery of ASD-associated behavior phenotypes such as repetitive behavior, anxiety-like phenotype, social preference, locomotor activity and digging behavior. We observed that *Ko* rats several behavior deficits congruent with an ASD-like phenotype, suggesting the critical role of mGluR5 signaling in determining increased susceptibility to autism.

Aberrant self-grooming has been associated with ASD-like phenotype in animal models representing stereotyped repetitive behavior [74, 78, 83]. Self-grooming behaviors in rats often include licking the body, paws/legs, genital area, and tail, as well as using their paws to wipe the face, ears, and head [73, 84]. Although self-grooming is a typical animal or rodent behavior performed for seconds to minutes, it is often flagged as abnormal when done more frequently or for an extended amount of time [85]. A number of studies have associated exaggerated self-grooming with ASD-like phenotype. The valproic acid (VPA)-exposed rat offspring have been shown to spend significantly more time self-grooming than control rats, suggesting ASD-related stereotyped behavior [73, 85]. Furthermore, di-(2-ethylhexyl) phthalate (DEHP) exposure has been shown to result in autism-like behavior, illustrated in part by the increased self-grooming time, which is comparable to the elevated duration observed in VPA rats [86]. Our results are in agreement with these studies as we observed that the *Ko* rats self-groomed significantly more frequently and for longer durations than the WT animals, thus exhibiting ASD-like phenotype.

Marble burying test is commonly used to determine the repetitive and anxiety-like phenotype, which is one of the most important hallmarks of ASD. Both the number of the marbles buried, as well as the amount of time the animal spends exploring the marbles or digging are often studied, similar to the type of analyses performed in our study. We observed that *Ko* rats buried more marbles and spent more time in digging the marbles compared to WT animals showing repetitive and anxiety-like behavior. On par with these findings, increased marble burying has been observed in various ASD animal models. The loss of *Tsc2* in Purkinje cells and deficiency of cyclooxygenase-2 (COX2) have been associated with ASD-like phenotype in rodent models [76, 87, 88]. Similarly, in a VPA induced ASD model, increased marble burying was observed that correlated with repetitive and anxiety-like behavior, mimicking clinical manifestations of ASD in human subjects [89].

An important parameter in the open-field test is the total distance travelled. If there are no differences in total distance traversed between different strains, it facilitates making valid comparisons for various behavior tests, as locomotor activity is no longer a confounding variable in the data analysis. The significant differences in locomotor activity may skew the data by preventing analyses for time spent in certain designated zones of the maze as differences may be due to inactivity instead of due to the genotype effect. In the present study, we observed no statistically significant difference in the locomotor activity of the *Ko* and WT rats. Therefore, we determined whether there are differences in the time spent in the center and periphery zones between the Ko and WT rats in the open-field test. However, we observed no significant differences in the time spent in the different zones. These results are in agreement with previous findings where no perimeter preference was observed in other animal models of ASD [76]. It is possible that the brain regions involved in this perimeter preference are not affected following the genetic ablation of mGluR5 (*Grm5*), which needs to be explored in future studies.

Impairments in social behavior is one of the characteristic hallmarks that is observed in individuals with ASD. mGluR5 signaling has been shown to play an important role in social interaction. As a proof of this concept, the mGluR5 positive allosteric modulator (PAM) CDPPB has been demonstrated to ameliorate social interaction deficits in the *Shank2*, *Shank3*, and *Sarm1 Ko* mouse models of ASD [59, 90, 91]. In addition, mGluR5 PAM [3-cyano-N-(1,3-diphenyl-1H-pyrazol-5-yl) benzamide] (CDPPB) attenuated social behavior deficits in *Sarm1* knock-out mouse model of ASD and prevented ASD-like alterations in rats exposed to cannabinoid during the prenatal period [91, 92]. These improvements in social behavior were attributed to the enhancement of mGluR5 levels. Our results are in agreement with these studies as mGluR5 *Ko* rats exhibited sociability deficits as well as impaired social behavior thus emphasizing the importance of mGluR5signaling in normal brain functioning.

In addition, our findings are in agreement with the data obtained from mGluR5 *Ko* mice. It has been shown that mGluR5 *ko* mice show impairments in social interaction and altered marble burying activity compared to WT controls [93]. However, no statistically significant differences were observed in self-grooming pattern and rotarod performance in mGluR5 *Ko* mice. Interestingly, mGluR5 *Ko* mice showed altered locomotor activity which may have confounded behavioral measurements. We do not observe such alteration in locomotor activity of *Ko* rats compared to WT animals thus suggesting this rat model can be used to understand the molecular underpinnings of ASD as well as testing the efficacy of future novel therapeutics for autism.

In summary, our findings suggest that mGluR5 *Ko* rats display ASD-like phenotype. Our results highlight the crucial role of mGluR5 signaling in ASD-associated behavior deficits. One of the limitations of our study is the constitutive deletion of mGluR5 expression. Future studies using conditional *Ko* rats with deletion of mGluR5 only in the brain or specific areas of the brain can shed further light on the role of mGluR5 signaling in the pathophysiology of ASD.

## References

[1] Lord C, Elsabbagh M, Baird G, Veenstra-Vanderweele J. Autism spectrum disorder. Lancet. 2018; 392(10146):508–520. doi: 10.1016/S0140-6736(18)31129-2 30078460 PMC7398158

[2] Bottema-Beutel K. Glimpses into the blind spot: Social interaction and autism. Journal of communication disorders. 2017; 68:24–34. doi: 10.1016/j.jcomdis.2017.06.008 28644991

[3] Bonnet-Brilhault F. Autism: An early neurodevelopmental disorder. Archives of Pediatrics. 2017; 24(4):384–390. 10.1016/j.arcped.2017.01.014 28256376

[4] Lai MC, Lombardo MV, Baron-Cohen S. Autism. Lancet. 2014; 383 (9920): 896–910. 10.1016/s0140-6736(13)61539-1. 24074734

[5] Maenner MJ, Shaw KA, Bakian AV, Bilder DA, Durkin MS, Esler A, et al. Prevalence and Characteristics of Autism Spectrum Disorder Among Children Aged 8 Years—Autism and Developmental Disabilities Monitoring Network, 11 Sites, United States, 2018. Morbidity and mortality weekly report. Surveillance summaries. 2021;70(11):1–16. doi: 10.15585/mmwr.ss7011a1 34855725 PMC8639024

[6] Purpura G, Costanzo V, Chericoni N, Puopolo M, Scattoni ML, Muratori F, et al. Bilateral patterns of repetitive movements in 6- to 12-month-old infants with autism spectrum disorders. Frontiers in psychology. 2017; 8:1168. doi: 10.3389/fpsyg.2017.01168 28744250 PMC5504227

[7] Wang Q, Hu Y, Shi D, Zhang Y, Zou X, Li S, et al. Children with autism spectrum disorder prefer looking at repetitive movements in a preferential looking paradigm Journal of autism and developmental disorders. 2018; 48(8):2821–2831. 10.1007/s10803-018-3546-5 29589273

[8] Muskens JB, Velders FP, Staal WG. Medical comorbidities in children and adolescents with autism spectrum disorders and attention deficit hyperactivity disorders: a systematic review. European child & adolescent psychiatry. 2017; 26(9):1093–1103. doi: 10.1007/s00787-017-1020-0 28674760 PMC5591355

[9] Crawley JN. Translational animal models of autism and neurodevelopmental disorders. Dialogues in clinical neuroscience. 2012; 14(3):293–305 doi: 10.31887/DCNS.2012.14.3/jcrawley 23226954 PMC3513683

[10] Haratizadeh S, Parvan M, Mohammadi S, Shabani M, Nozari M. An overview of modeling and behavioral assessment of autism in the rodent. International journal of developmental neuroscience: the official journal of the International Society for Developmental Neuroscience. 2021;81(3):221–228. doi: 10.1002/jdn.10096 33570815

[11] Strekalova T, Svirin E, Veniaminova E, Kopeikina E, Veremeyko T, Yung AWY, et al. ASD-like behaviors, a dysregulated inflammatory response and decreased expression of PLP1 characterize mice deficient for sialyltransferase ST3GAL5. Brain, behavior, & immunity—health. 2021;16:100306. 10.1016/j.bbih.2021.100306 PMC8474501 34589798

[12] Kazdoba TM, Leach PT, Crawley JN. Behavioral phenotypes of genetic mouse models of autism. Genes, brain, and behavior. 2016; 15(1):7–26. doi: 10.1111/gbb.12256 26403076 PMC4775274

[13] Sungur AÖ, Schwarting RKW, Wöhr M. Behavioral phenotypes and neurobiological mechanisms in the Shank1 mouse model for autism spectrum disorder: A translational perspective. Behavioural brain research. 2018; 352:46–61. doi: 10.1016/j.bbr.2017.09.038 28963042

[14] Kazdoba TM, Leach PT, Yang M, Silverman JL, Solomon M, Crawley JN. Translational Mouse Models of Autism: Advancing Toward Pharmacological Therapeutics. Current Topics in Behavioral Neurosciences. 2016; 28:1–52. doi: 10.1007/7854_2015_5003 27305922 PMC5116923

[15] Dhamne SC, Silverman JL, Super CE, Lammers SHT, Hameed MQ, Modi ME, et al. Replicable in vivo physiological and behavioral phenotypes of the Shank3B null mutant mouse model of autism. Molecular Autism. 2017; 8:26. doi: 10.1186/s13229-017-0142-z 28638591 PMC5472997

[16] Vanderschuren LJ, Achterberg EJ, Trezza V. The neurobiology of social play and its rewarding value in rats. Neuroscience and biobehavioral reviews. 2016; 70:86–105 doi: 10.1016/j.neubiorev.2016.07.025 27587003 PMC5074863

[17] Leite IS, Castelhano AS, Cysneiros RM. Effect of diazepam on sociability of rats submitted to neonatal seizures. Data Brief. 2016; 7:686–91. doi: 10.1016/j.dib.2016.03.029 27054178 PMC4802817

[18] Vanderschuren LJ, Niesink RJ, Van Ree JM. The neurobiology of social play behavior in rats. Neurosci Biobehav Rev. 1997; 21(3):309–26. doi: 10.1016/s0149-7634(96)00020-6 9168267

[19] Pellis SM, Pellis VC. Play fighting of rats in comparative perspective: A schema for neurobehavioral analyses. Neurosci Biobehav Rev. 1998; 23:87–101. doi: 10.1016/s0149-7634(97)00071-7 9861614

[20] Carnevali L, Montano N, Statello R, Coudé G, Vacondio F, Rivara S, et al. Social stress contagion in rats: Behavioural, autonomic and neuroendocrine correlates. Psychoneuroendocrinology. 2017; 82:155–163. doi: 10.1016/j.psyneuen.2017.05.017 28550792

[21] Castro GP, Medeiros DC, Guarnieri LO, Mourão FAG, Pinto HPP, Pereira GS, et al. Wistar audiogenic rats display abnormal behavioral traits associated with artificial selection for seizure susceptibility. Epilepsy Behav. 2017; 71(Pt B):243–249. doi: 10.1016/j.yebeh.2015.08.039 26440280

[22] Branchi I, Santucci D, Alleva E. Ultrasonic vocalisation emitted by infant rodents: a tool for assessment of neurobehavioural development. Behavioural Brain Research 2001; 125:49–56. doi: 10.1016/s0166-4328(01)00277-7 11682093

[23] Brudzynski SM. Ethotransmission: communication of emotional states through ultrasonic vocalization in rats. Current Opinion in Neurobiology 2013; 23:310–7. doi: 10.1016/j.conb.2013.01.014 23375168

[24] Ehret G. Infant rodent ultrasounds—a gate to the understanding of sound communication. Behavior Genetics 2005; 35:19–22. doi: 10.1007/s10519-004-0853-8 15674530

[25] Hofer MA, Shair HN. Ultrasonic vocalization, laryngeal braking, and thermogenesis in rat pups: a reappraisal. Behavioral Neuroscience 1993; 107:354–62. doi: 10.1037//0735-7044.107.2.354 8484900

[26] Knutson B, Burgdorf J, Panksepp J. Ultrasonic vocalizations as indices of affective states in rats. Psychological Bulletin 2002; 128:961–77. doi: 10.1037/0033-2909.128.6.961 12405139

[27] Knutson B, Burgdorf J, Panksepp J. Anticipation of play elicits high-frequency ultrasonic vocalizations in young rats. Journal of Comparative Psychology 1998; 112:65–73. doi: 10.1037/0735-7036.112.1.65 9528115

[28] De Rubeis S, Buxbaum JD. Genetics and genomics of autism spectrum disorder: embracing complexity. Human Molecular Genetics. 2015; 24(R1):R24–31. doi: 10.1093/hmg/ddv273 26188008 PMC4675826

[29] Wu N, Wang Y, Jia JY, Pan YH, Yuan XB. Association of CDH11 with Autism Spectrum Disorder Revealed by Matched-gene Co-expression Analysis and Mouse Behavioral Studies. Neuroscience Bulletin. 2022;38(1):29–46. doi: 10.1007/s12264-021-00770-0 34523068 PMC8783018

[30] Viggiano M, D’Andrea T, Cameli C, Posar A, Visconti P, Scaduto MC, et al. Contribution of CACNA1H Variants in Autism Spectrum Disorder Susceptibility. Frontiers in Psychiatry. 2022;13:858238. doi: 10.3389/fpsyt.2022.858238 35350424 PMC8957782

[31] Gaugler T, Klei L, Sanders SJ, Bodea CA, Goldberg AP, Lee AB, et al. Most genetic risk for autism resides with common variation. Nature Genetics. 2014; 46(8):881–5. doi: 10.1038/ng.3039 25038753 PMC4137411

[32] Klei L, Sanders SJ, Murtha MT, Hus V, Lowe JK, Willsey AJ, et al. Common genetic variants, acting additively, are a major source of risk for autism. Molecular Autism. 2012; 3(1):9. doi: 10.1186/2040-2392-3-9 23067556 PMC3579743

[33] Fanjul-Fernández M, Brown NJ, Hickey P, Diakumis P, Rafehi H, Bozaoglu K, et al. A family study implicates GBE1 in the etiology of autism spectrum disorder. Hum Mutation. 2022;43(1):16–29. doi: 10.1002/humu.24289 34633740 PMC8720068

[34] Carducci F, Ardiccioni C, Fiorini R, Vignini A, Di Paolo A, Alia S, et al. The ALA5/ALA6/ALA7 repeat polymorphisms of the glutathione peroxidase-1 (GPx1) gene and autism spectrum disorder. Autism research: official journal of the International Society for Autism Research. 2022;15(2):215–221 doi: 10.1002/aur.2655 34997988 PMC9304179

[35] Marques AR, Santos JX, Martiniano H, Vilela J, Rasga C, Romão L, et al. Gene Variants Involved in Nonsense-Mediated mRNA Decay Suggest a Role in Autism Spectrum Disorder. Biomedicines. 2022;10(3):665. doi: 10.3390/biomedicines10030665 35327467 PMC8945030

[36] Tzanoulinou S, Musardo S, Contestabile A, Bariselli S, Casarotto G, Magrinelli E, et al. Inhibition of Trpv4 rescues circuit and social deficits unmasked by acute inflammatory response in a Shank3 mouse model of Autism. Molecular psychiatry. 2022 (in press). 10.1038/s41380-021-01427-0 PMC9126815 35022531

[37] Monteiro P, Feng G. SHANK proteins: roles at the synapse and in autism spectrum disorder. Nature reviews. Neuroscience. 2017; 18(3):147–157. 28179641 10.1038/nrn.2016.183

[38] Kanani F, Study D, Balasubramanian M. SHANK3 variant as a cause of nonsyndromal autism in an 11-year-old boy and a review of published literature. Clinical dysmorphology. 2018; 27(4):113–115. doi: 10.1097/MCD.0000000000000232 29939863

[39] Leblond CS, Nava C, Polge A, Gauthier J, Huguet G, Lumbroso S, et al. Meta-analysis of SHANK Mutations in Autism Spectrum Disorders: a gradient of severity in cognitive impairments. PLoS Genetics. 2014; 10(9):e1004580. doi: 10.1371/journal.pgen.1004580 25188300 PMC4154644

[40] Uchino S, Waga C. SHANK3 as an autism spectrum disorder-associated gene. Brain & development. 2013; 35(2):106–10. doi: 10.1016/j.braindev.2012.05.013 22749736

[41] Ito H, Morishita R, Nagata KI. Autism spectrum disorder-associated genes and the development of dentate granule cells. Medical molecular morphology. 2017; 50(3):123–129. doi: 10.1007/s00795-017-0161-z 28534217

[42] Gonzales ML, LaSalle JM. The role of MeCP2 in brain development and neurodevelopmental disorders. Current psychiatry reports. 2010; 12(2):127–34. doi: 10.1007/s11920-010-0097-7 20425298 PMC2847695

[43] Liu Z, Li X, Zhang JT, Cai YJ, Cheng TL, Cheng C, et al. Autism-like behaviours and germline transmission in transgenic monkeys overexpressing MeCP2. Nature. 2016; 530(7588):98–102. doi: 10.1038/nature16533 26808898

[44] Jamain S, Quach H, Betancur C, Råstam M, Colineaux C, Gillberg IC, et al. Mutations of the X-linked genes encoding neuroligins NLGN3 and NLGN4 are associated with autism. Nature Genetics. 2003; 34(1):27–9. doi: 10.1038/ng1136 12669065 PMC1925054

[45] Xu X, Xiong Z, Zhang L, Liu Y, Lu L, Peng Y, et al. Variations analysis of NLGN3 and NLGN4X gene in Chinese autism patients. Molecular biology reports. 2014; 41(6):4133–40. doi: 10.1007/s11033-014-3284-5 24570023

[46] Kasem E, Kurihara T, Tabuchi K. Neurexins and neuropsychiatric disorders. Neuroscience Research. 2018; 127:53–60. doi: 10.1016/j.neures.2017.10.012 29221905

[47] Onay H, Kacamak D, Kavasoglu AN, Akgun B, Yalcinli M, Kose S, et al. Mutation analysis of the NRXN1 gene in autism spectrum disorders. Balkan journal of medical genetics: BJMG. 2017; 19(2):17–22. doi: 10.1515/bjmg-2016-0031 28289584 PMC5343326

[48] Pugin A, Faundes V, Santa María L, Curotto B, Aliaga S, et al. Clinical, molecular, and pharmacological aspects of FMR1 related disorders. Neurologia. 2017; 32(4):241–252. 25529181 10.1016/j.nrl.2014.10.009

[49] Liu Y, Hu Z, Xun G, Peng Y, Lu L, et al. Mutation analysis of the NRXN1 gene in a Chinese autism cohort. Journal of psychiatric research. 2012; 46(5):630–4. doi: 10.1016/j.jpsychires.2011.10.015 22405623

[50] Jung KM, Sepers M, Henstridge CM, et al. Uncoupling of the endocannabinoid signalling complex in a mouse model of fragile X syndrome. Nat Commun. 2012;3:1080. doi: 10.1038/ncomms2045 23011134 PMC3657999

[51] Zantomio D, Chana G, Laskaris L, Testa R, Everall I, Pantelis C, et al. Convergent evidence for mGluR5 in synaptic and neuroinflammatory pathways implicated in ASD. Neuroscience and biobehavioral reviews. 2015; 52:172–7. doi: 10.1016/j.neubiorev.2015.02.006 25704074

[52] Mao LM, Bodepudi A, Chu XP, Wang JQ. Group I Metabotropic Glutamate Receptors and Interacting Partners: An Update International journal of molecular sciences. 2022;23(2):840. 10.3390/ijms23020840 PMC8778124 35055030

[53] Acher FC, Cabayé A, Eshak F, Goupil-Lamy A, Pin JP. Metabotropic glutamate receptor orthosteric ligands and their binding sites. Neuropharmacology. 2022;204:108886. doi: 10.1016/j.neuropharm.2021.108886 34813860

[54] Awad H, Hubert GW, Smith Y, Levey AI, Conn PJ. Activation of metabotropic glutamate receptor 5 has direct excitatory effects and potentiates NMDA receptor currents in neurons of the subthalamic nucleus. The Journal of neuroscience: the official journal of the Society for Neuroscience. 2000; 20: 7871–9. doi: 10.1523/JNEUROSCI.20-21-07871.2000 11050106 PMC6772731

[55] Jong YJ, Kumar V, O’Malley KL. Intracellular metabotropic glutamate receptor 5 (mGluR5) activates signaling cascades distinct from cell surface coun-terparts. The Journal of biological chemistry. 2009; 284: 35827–38. doi: 10.1074/jbc.M109.046276 19840937 PMC2791012

[56] Abushik PA, Niittykoski M, Giniatullina R, Shakirzyanova A, Bart G, Fayuk D, et al. The role of NMDA and mGluR5 receptors in calcium mobilization and neurotoxicity of homocysteine in trigem-inal and cortical neurons and glial cells. Journal of neurochemistry. 2014; 129: 264–74. doi: 10.1111/jnc.12615 24266734

[57] Panatier A, Robitaille R. Astrocytic mGluR5 and the tripartite synapse. Neuroscience. 2016; 323:29–34. doi: 10.1016/j.neuroscience.2015.03.063 25847307

[58] Potter WB, Basu T, O’Riordan KJ, Kirchner A, Rutecki P, Burger C, et al. Reduced juvenile long-term depression in tuberous sclerosis complex is mitigated in adults by compensatory recruitment of mGluR5 and Erk signaling. PLoS Biology. 2013; 11: e1001627. doi: 10.1371/journal.pbio.1001627 23966835 PMC3742461

[59] Vicidomini C, Ponzoni L, Lim D, Schmeisser MJ, Reim D, Morello N, et al. Pharmacological enhancement of mGlu5 receptors rescues behavioral deficits in SHANK3 knock-out mice. Molecular Psychiatry. 2017; 22(5):784. doi: 10.1038/mp.2016.70 27113996

[60] Chana G, Laskaris L, Pantelis C, Gillett P, Testa R, Zantomio D, et al. Decreased expression of mGluR5 within the dorsolateral prefrontal cortex in autism and increased microglial number in mGluR5 knockout mice: Pathophysiological and neurobehavioral implications. Brain, behavior, and immunity. 2015; 49:197–205. doi: 10.1016/j.bbi.2015.05.009 26052099

[61] Skafidas E, Testa R, Zantomio D, Chana G, Everall IP, Pantelis C. Predicting the diagnosis of autism spectrum disorder using gene pathway analysis. Molecular Psychiatry. 2014; 19(4):504–10. doi: 10.1038/mp.2012.126 22965006 PMC3966080

[62] Koros E, Rosenbrock H, Birk G, Weiss C, Sams-Dodd F. The selective mGlu5 receptor antagonist MTEP, similar to NMDA receptor antagonists, induces social isolation in rats. Neuropsychopharmacology. 2007; 32(3):562–76. doi: 10.1038/sj.npp.1301133 16794564

[63] Gogliotti RG, Senter RK, Rook JM, Ghoshal A, Zamorano R, Malosh C, et al. mGlu5 positive allosteric modulation normalizes synaptic plasticity defects and motor phenotypes in a mouse model of Rett syndrome. Human molecular genetics. 2016; 25(10):1990–2004. doi: 10.1093/hmg/ddw074 26936821 PMC5062588

[64] Jin XR, Chen XS, Xiao L. MeCP2 Deficiency in Neuroglia: New Progress in the Pathogenesis of Rett Syndrome. Frontiers in Molecular Neuroscience. 2017; 10:316. doi: 10.3389/fnmol.2017.00316 29046627 PMC5632713

[65] Moretti P, Levenson JM, Battaglia F, Atkinson R, Teague R, Antalffy B, et al. Learning and memory and synaptic plasticity are impaired in a mouse model of Rett syndrome. The Journal of neuroscience: the official journal of the Society for Neuroscience. 2006; 26(1):319–27. 16399702 10.1523/JNEUROSCI.2623-05.2006 PMC6674314

[66] Jiménez JA, Zylka MJ. Controlling litter effects to enhance rigor and reproducibility with rodent models of neurodevelopmental disorders. J Neurodevelop Disord. 2021; 13: 2. doi: 10.1186/s11689-020-09353-y 33397279 PMC7780384

[67] Lazic SE, Essioux L. Improving basic and translational science by accounting for litter-to-litter variation in animal models. BMC Neurosci. 2013; 14: 37. doi: 10.1186/1471-2202-14-37 23522086 PMC3661356

[68] Pan T, Jiang C, Cheng J, Xie J, Liu X, Xu W, et al. Autism-Like Behavior in the Offspring of CYP11A1-Overexpressing Pregnant Rats. Frontiers in Neuroscience. 2021;15:774439. doi: 10.3389/fnins.2021.774439 35002603 PMC8733305

[69] Sethi S, Keil Stietz KP, Valenzuela AE, Klocke CR, Silverman JL, et al. Developmental Exposure to a Human-Relevant Polychlorinated Biphenyl Mixture Causes Behavioral Phenotypes That Vary by Sex and Genotype in Juvenile Mice Expressing Human Mutations That Modulate Neuronal Calcium. Frontiers in Neuroscience. 2021;15:766826. doi: 10.3389/fnins.2021.766826 34938155 PMC8685320

[70] Berg EL, Pedersen LR, Pride MC, et al. Developmental exposure to near roadway pollution produces behavioral phenotypes relevant to neurodevelopmental disorders in juvenile rats. Transl Psychiatry. 2020;10(1):289. doi: 10.1038/s41398-020-00978-0 32807767 PMC7431542

[71] Oroszi T, Geerts E, de Boer SF, Schoemaker RG, van der Zee EA, Nyakas C. Whole Body Vibration Improves Spatial Memory, Anxiety-Like Behavior, and Motor Performance in Aged Male and Female Rats. Frontiers in aging neuroscience. 2022;13:801828. doi: 10.3389/fnagi.2021.801828 35126091 PMC8815031

[72] Leal PEPT, da Silva AA, Rocha-Gomes A, Riul TR, Cunha RA, Reichetzeder C, et al. High-Salt Diet in the Pre- and Postweaning Periods Leads to Amygdala Oxidative Stress and Changes in Locomotion and Anxiety-Like Behaviors of Male Wistar Rats. Frontiers in behavioral neuroscience. 2022;15:779080. doi: 10.3389/fnbeh.2021.779080 35058757 PMC8763963

[73] Gu Y, Han Y, Ren S, Zhang B, Zhao Y, Wang X, et al. Correlation among gut microbiota, fecal metabolites and autism-like behavior in an adolescent valproic acid-induced rat autism model. Behavioural brain research. 2022;417:113580. doi: 10.1016/j.bbr.2021.113580 34555431

[74] Kalueff AV, Stewart AM, Song C, Berridge KC, Graybiel AM, Fentress JC. Neurobiology of rodent self-grooming and its value for translational neuroscience. Nature reviews. Neuroscience 2016;17(1):45–59. 10.1038/nrn.2015.8 PMC4840777 26675822

[75] Hamilton SM, Green JR, Veeraragavan S, Yuva L, McCoy A, Wu Y, et al. Fmr1 and Nlgn3 knockout rats: novel tools for investigating autism spectrum disorders. Behavioral Neuroscience. 2014;128(2):103–9. doi: 10.1037/a0035988 24773431

[76] Scott KE, Kazazian K, Mann RS, Möhrle D, Schormans AL, Schmid S, et al. Loss of Cntnap2 in the Rat Causes Autism-Related Alterations in Social Interactions, Stereotypic Behavior, and Sensory Processing. Autism Research. 2020;13(10):1698–1717. doi: 10.1002/aur.2364 32918359

[77] Deng W, Ke H, Wang S, Li Z, Li S, Lv P, et al. Metformin Alleviates Autistic-Like Behaviors Elicited by High-Fat Diet Consumption and Modulates the Crosstalk Between Serotonin and Gut Microbiota in Mice. Behavioural neurology. 2022;2022:6711160. doi: 10.1155/2022/6711160 35222739 PMC8872653

[78] Angoa-Pérez M, Kane MJ, Briggs DI, Francescutti DM, Kuhn DM. Marble burying and nestlet shredding as tests of repetitive, compulsive-like behaviors in mice. Journal of Visualized Experiments: JoVE. 2013 Dec 24;(82):50978. doi: 10.3791/50978 24429507 PMC4108161

[79] Kim H, Lim CS, Kaang BK. Neuronal mechanisms and circuits underlying repetitive behaviors in mouse models of autism spectrum disorder. Behavioral and brain functions: BBF. 2016;12(1):3. doi: 10.1186/s12993-016-0087-y 26790724 PMC4719705

[80] Silverman JL, Yang M, Lord C, Crawley JN. Behavioural phenotyping assays for mouse models of autism. Nature Reviews Neuroscience. 2010;11(7):490–502. doi: 10.1038/nrn2851 20559336 PMC3087436

[81] Sturman O., Germain P.-L., & Bohacek J. (2018). Exploratory rearing: A context- and stress-sensitive behavior recorded in the open-field test. Stress (Amsterdam, Netherlands), 21(5), 443–452. doi: 10.1080/10253890.2018.1438405 29451062

[82] Thirtamara Rajamani K, Doherty-Lyons S, Bolden C, et al. Prenatal and early-life exposure to high-level diesel exhaust particles leads to increased locomotor activity and repetitive behaviors in mice. Autism Res. 2013;6(4):248–257. doi: 10.1002/aur.1287 23495194 PMC8935356

[83] Ku KM, Weir RK, Silverman JL, Berman RF, Bauman MD. Behavioral phenotyping of juvenile Long-Evans and Sprague-Dawley rats: Implications for preclinical models of autism spectrum disorders. PLoS One. 2016; 11(6):e0158150. doi: 10.1371/journal.pone.0158150 27351457 PMC4924796

[84] Kirsten TB, Bernardi MM. Prenatal lipopolysaccharide induces hypothalamic dopaminergic hypoactivity and autistic-like behaviors: Repetitive self-grooming and stereotypies. Behav Brain Res. 2017;331:25–29. doi: 10.1016/j.bbr.2017.05.013 28526515

[85] Du L, Zhao G, Duan Z, Li F. Behavioral improvements in a valproic acid rat model of autism following vitamin D supplementation. Psychiatry Res. 2017;253:28–32. doi: 10.1016/j.psychres.2017.03.003 28324861

[86] Li Y, Zhao Y, Lu Y, et al. Autism spectrum disorder-like behavior induced in rat offspring by perinatal exposure to di-(2-ethylhexyl) phthalate [published online ahead of print, 2022 Mar 7]. Environ Sci Pollut Res Int. 2022; doi: 10.1007/s11356-022-19531-1 35254616

[87] Wong CT, Bestard-Lorigados I, Crawford DA. Autism-related behaviors in the cyclooxygenase-2-deficient mouse model. Genes Brain Behav. 2019;18(1):e12506. doi: 10.1111/gbb.12506 30027581

[88] Reith RM, McKenna J, Wu H, et al. Loss of Tsc2 in Purkinje cells is associated with autistic-like behavior in a mouse model of tuberous sclerosis complex. Neurobiol Dis. 2013;51:93–103. doi: 10.1016/j.nbd.2012.10.014 23123587

[89] Choi CS, Gonzales EL, Kim KC, et al. The transgenerational inheritance of autism-like phenotypes in mice exposed to valproic acid during pregnancy. Sci Rep. 2016;6:36250. doi: 10.1038/srep36250 27819277 PMC5098241

[90] Won H, Lee HR, Gee HY, Mah W, Kim JI, Lee J, et al. Autistic-like social behaviour in Shank2-mutant mice improved by restoring NMDA receptor function. Nature. 2012;486(7402):261–5. doi: 10.1038/nature11208 22699620

[91] Lin CW, Chen CY, Cheng SJ, Hu HT, Hsueh YP. Sarm1 deficiency impairs synaptic function and leads to behavioral deficits, which can be ameliorated by an mGluR allosteric modulator. Frontiers in cellular neuroscience. 2014;8:87. doi: 10.3389/fncel.2014.00087 24744698 PMC3978259

[92] Bara A, Manduca A, Bernabeu A, Borsoi M, Serviado M, Lassalle O, et al. Sex-dependent effects of in utero cannabinoid exposure on cortical function. Elife. 2018;7:e36234. doi: 10.7554/eLife.36234 30201092 PMC6162091

[93] Xu J, Marshall JJ, Kraniotis S, Nomura T, Zhu Y, Contractor A. Genetic disruption of Grm5 causes complex alterations in motor activity, anxiety and social behaviors. Behav Brain Res. 2021;411:113378. doi: 10.1016/j.bbr.2021.113378 34029630 PMC8238894

